# Timely Words

**Published:** 1897-08

**Authors:** 


TIMELY WORDS.—Dr. Paschall, of San Antonio, has a paper in this issue of the JOURNAL, on dentition and diarrhœa. As this is the season of the greatest mortality among infants, his words will be read with profit. The JOURNAL differs with the doctor in his estimate of substitutes for mother's milk, and can recommend at least one article: Mellin's food. We would also advise Fairchild's peptonizing lubis, and suggest that interested parties write to Mr. Fairchild (Fairchild Bros. & Forlis, New York) for his little book on digestive ferments.

---

AS A TRIP TO MEXICO seems now to be the proper fad, our readers who contemplate making the trip, will do well to make a memorandum of our recent experiences and observations, and profit by them. If one knew just where to go and what to do on arrival, he would save a good deal of money and embarrassment. At first we took our meals a la carte at the Maison Doree, a high priced French restaurant, and we paid as much for a single meal as a day's board later cost the two of us. There were a number of Texans in the city, and we soon "got on" to the right thing. Board and rooms are the cheapest things we found there. Everything else is about the same price as in Ausin, the difference in the money considered; that is, double the price in American money.

To those who contemplate a trip to the City of Mexico, we would say: first; go by way of Laredo, by all means; it is 256 miles the shortest route, and the trip is made in exactly fifty hours, two nights in the sleeper, costing, from Laredo to Mexico, $9.00, Mexican. The I. & G. N. R. R., the only road to Laredo, is strictly first-class in every respect; the road bed is solid and smooth and level, the rails of steel, and the trains fairly glide over them like a skater on ice—except that between San Antonio and Laredo the temperature is anything but suggestive of ice. We never saw more handsomely equipped trains; the Palace and Buffet cars are a luxury; any one can have excellent meals served at his seat in the car, excellent and clean,—almost anything one could want. The management is well disciplined, and travelers receive every attention and comfort. At Laredo the train crosses the river, where the custom officials examine one's baggage—the railroad officials aiding the stranger who does not speak the Spanish language. The only change of cars from Austin to City of Mexico is made here. Here you enter the snug little sleepers of the Mexican National road, and at once feel equally at home; everything good to eat is served on board by the most polite and attentive stewards; the beds are clean and comfortable, and the train rolls rapidly and without a jolt or swing. On arrival in the city direct the bus driver to 13 San Juan de Letran. It is near the corner of San Francisco street, the principal thoroughfare, and in the center of business; near Iturbide and the Jardin hotels; near the general offices of all railroads; near the club house of the American club, just opposite which is a place where you can buy stamps and mail letters without having to go to the postoffice. Here Mrs. Hopkins, an excellent lady from California, will rent you a nicely, newly furnished room, with towels and the services of a man servant, for $1 a day, Mexican money, for one room, or $2 a day for two rooms. At the Jardin hotel (pronounced "Hardeen") one can get good table d 'hote board for $1.50 a day, Mexican money. Mr. Wise, late of San Antonio, has a restaurant at 13½ San Juan de Letran, just under Mrs. Hopkins' establishment, where good meals are served at $1.20 a day.

Tourists will do well to heed the advice given us by Dr. Bibb. Be careful what kind of water you drink. He has all his drinking water boiled. We drank filtered water and seltzer by default. There is a great deal of typhoid fever there, the doctor says; and one cannot be too careful of "germs." He says the strawberries, tomatoes, lettuce, etc., are irrigated with ditch water, and are, therefore, a source of great danger, and should not be eaten raw.

It looks a little paradoxical in going into the Torrid zone in mid-summer, to carry an overcoat; yet one will need an overcoat. We wore ours every day, and flannel underwear also; for, notwithstanding it is between the Tropic of Cancer and the Equator, it must not be forgotten that the altitude of the city is 7,200 feet above the sea; and snow is in sight, part of the time. On a clear day the great volcano Popocatapetl can be seen covered with snow. It makes one shiver even now to recall our first glimpse of this snowclad peak in the middle of July. Every afternoon it rains, more or less, the entire summer; hence, the nights are cool and damp.

If you go to Mexico, go via Laredo,—take your flannels and overcoat. Stop at Mrs. Hopkins, if you want to feel at home, and get your meals at Wise's. There are no fireplaces, grates nor stoves in any of the houses, and if you are as thin blooded as yours truly you will suffer unless you take with you clothing suited to a cold and changeable climate.

Of the scenery on the line of the National, it is useless to attempt to speak. It is beyond the power of words to describe and must be seen to be appreciated.

